# THE EFFECT OF FRAILTY ON COGNITION IN OLDER ADULTS: THE MEDIATING ROLE OF DEPRESSIVE SYMPTOMS

**DOI:** 10.1093/geroni/igae098.2773

**Published:** 2024-12-31

**Authors:** Yiyang Yuan, Kate Lapane

**Affiliations:** University of Massachusetts Chan Medical School, Worcester, Massachusetts, United States; University of Massachusetts Chan Medical School, Worcester, Massachusetts, United States

## Abstract

This study aims to examine the effect of physical frailty on cognitive function with depressive symptoms as a mediator in older adults in the United States. The Health and Retirement Study (HRS 2016; baseline) was linked to its sub-study Harmonized Cognitive Assessment Protocol (HCAP; follow-up), which was conducted in a randomly selected half of the 2016 HRS participants, to identify 1,503 adults aged 65 and above. The exposure physical frailty and mediator depressive symptoms were measured in HRS at baseline respectively by the Fried frailty phenotype on low weight, physical inactivity, exhaustion, weakness, slowness (total score: 0-5; dichotomized as not frail (score: 0) vs. frail (score: 1-5)) and the Center for Epidemiological Studies Depression (CES-D) Scale (total score: 0-8). The outcome overall cognitive function was measured in HCAP at follow-up by the Mini-Mental State Examination (total score: 0-30). Causal mediation analysis was used to assess the direct effect of physical frailty and its indirect effect through depressive symptoms on cognitive function, considering the interaction between the exposure and the mediator. Having physical frailty would decrease cognitive function by a total effect of -0.26 (95% CI: -0.59 to 0.07), with total natural direct effect of -0.14 (95% CI: -0.48 to 0.20) and pure natural indirect effect through depressive symptoms of -0.12 (95% CI: -0.22 to -0.01). The lack of clinically meaningful effects is likely due to the short length of follow-up. This study employs the natural temporal order in HRS and HCAP, but analysis with longer follow-up is warranted.

